# Duration of humoral immunity from smallpox vaccination and its cross-reaction with Mpox virus

**DOI:** 10.1038/s41392-023-01574-6

**Published:** 2023-09-15

**Authors:** Entao Li, Xiaoping Guo, Dongxiang Hong, Qizan Gong, Wenyu Xie, Tingting Li, Jian Wang, Xia Chuai, Sandra Chiu

**Affiliations:** 1 Department of Laboratory Medicine, The First Affiliated Hospital of USTC, Division of Life Sciences and Medicine, University of Science and Technology of China, Hefei, Anhui China; 2 Division of Life Sciences and Medicine, University of Science and Technology of China, Hefei, Anhui China; 3 Department of Clinical Laboratory, First Affiliated Hospital of Anhui Medical University, Hefei, Anhui China; 4 State Key Laboratory of Virology, Wuhan Institute of Virology, Center for Biosafety Mega Science, Chinese Academy of Sciences, Wuhan, Hubei China; 5 Core Unit of National Clinical Research Center for Laboratory Medicine, Hefei, Anhui China; 6 Key Laboratory of Anhui Province for Emerging and Reemerging Infectious Diseases, Hefei, Anhui China

**Keywords:** Infectious diseases, Infectious diseases

## Abstract

The ongoing pandemic caused by mpox virus (MPXV) has become an international public health emergency that poses a significant threat to global health. The vaccinia virus Tiantan strain (VTT) was used to vaccinate against smallpox in China 42 years ago. It is urgent to assess the level of immunity to smallpox in individuals vaccinated 43 or more years ago and evaluate their immunological susceptibility to MPXV. Here, we recruited 294 volunteers and detected the level of residual humoral immunity, including the vaccinia-specific IgG level and neutralizing antibody titer, and the cross-antibodies of MPXV A29L, B6R, A35R, and M1R. Our results showed that the humoral immunity from the smallpox vaccine in the population still remains, and VTT-specific NAb levels wane with age. The majority of the population pre-1981 who should be immunized with VTT still maintains certain levels of MPXV-specific antibodies, in particular, targeting A35R and B6R antigens. Furthermore, we separately analyzed the correlations between the OD450 values of VTT-specific IgG and A35R-specific IgG, B6R-specific IgG, and A29L-specific IgG with plasma samples diluted 1:40, showing a linear correlation (*p* < 0.0001). Our findings suggest that most Chinese populations still maintain VTT-specific IgG antibodies for 42 or more years after smallpox vaccination and could provide some level of protection against MPXV.

## Introduction

Mpox is a zoonotic disease caused by the mpox virus (MPXV) characterized by smallpox-like symptoms, including fever and rash, but the signs of mpox are milder and the mortality rate is lower than those of smallpox.^1^ The first mpox case was diagnosed in 1970 in the Democratic Republic of the Congo (DRC), and since then, it has been endemic in Central and West Africa over the last few decades.^2,3^ Occasional outbreaks outside Africa were reported in the United States, the United Kingdom (UK), Israel, and Singapore prior to 2022.^4–6^ However, since the first confirmed case was reported in the UK on May 6th 2022, mpox has quickly spread across many countries worldwide. The 2022 outbreak of mpox has drawn great attention because it is continually reported in hitherto nonendemic countries, and the World Health Organization (WHO) declared mpox a public health emergency of international concern on July 23rd 2022.^7^ As of June 21st 2023, a total of 88,026 cases of mpox have been reported across 111 countries, including 148 deaths, posing a greater global threat to humans.^8^

Vaccination is always the most effective approach to control and prevent mpox epidemics. MPXV belongs to the *Orthopoxvirus* genus of the *Poxviridae* family, which is genetically closely linked to other Orthopoxvirus viruses, such as variola virus (VARV), vaccinia virus (VACV) and cowpox virus (CPXV). Vaccination using VACV against smallpox has been shown to provide 85% protection against MPXV infection based on epidemiological features, as observed in Zaire in the past.^9^ However, following the global eradication of smallpox in 1980, routine smallpox vaccination was gradually discontinued in many countries.^10^ Few people born following the eradication of smallpox have received smallpox vaccination, making those younger than 43 years old vulnerable to other orthopoxviruses, such as MPXV and CPXV infections. Although previous studies have shown that vaccination-induced immunity has been maintained for more than three decades,^11^ immunity still wanes with time since vaccination.^12–14^ Therefore, the cessation of smallpox vaccination over the past decades has increased the risk of MPXV in humans.^15,16^ Currently, the two vaccines ACAM2000 and JYNNEOS, both initially developed for smallpox, are available to prevent mpox. ACAM2000, a replicating vaccinia virus-based second generation smallpox vaccine, can provide effective protection even after exposure to MPXV.^17,18^ However, ACAM2000 can often lead to severe adverse effects, and it is contraindicated in individuals with pregnancy, atopic dermatitis, or immune deficiencies.^19^ JYNNEOS is the 3^rd^ generation vaccine produced from the replication-deficient modified vaccinia Ankara-Bavarian Nordic (MVABN strain). It was approved by the US Food and Drug Administration (FDA) in 2019 to prevent both smallpox and mpox disease in individuals over 18 years of age considered to be at high risk for both infections.^20^ JYNNEOS has been shown to be safer than ACAM2000 due to its poor ability to replicate in human cells. However, both vaccines are not recommended for the general public,^21^ and they cannot completely protect against MPXV. Breakthrough infections have been found in people who received the smallpox vaccine after high-risk exposure to MPXV.^22,23^ Moreover, the current supplies of these two vaccines are limited, and they are not accessible to all countries.^24^ Thus, it is crucial to evaluate the susceptibility of MPXV in the general population and develop effective vaccination strategies against mpox.

In China, the vaccinia virus Tiantan strain (VTT) was historically used to vaccinate against smallpox.^25^ After the WHO recommended that routine smallpox vaccination be discontinued, the Chinese government stopped the national smallpox vaccination program in 1981. However, little is known about the duration and degree of residual immunity to smallpox in individuals vaccinated 43 or more years ago. Furthermore, mpox has never emerged in China before 2022, and due to the lack of live MPXV and animal infection models, whether the residual immunity of VTT can provide sufficient cross-protection in the event of MPXV exposure is unknown.^26^ Therefore, it is critical to assess the level of immunity to smallpox in individuals vaccinated 42 or more years ago and evaluate their immunological susceptibility to MPXV.

Antibody responses (both total IgG and neutralizing antibody) were reported to play crucial roles in protection against poxvirus diseases.^12,27^ MPXV has a large DNA genome that generates two antigenically distinct virion forms: intracellular mature virus (IMV) and extracellular enveloped virus (EEV), which are similar to other orthopoxviruses. The IMV surface proteins A29L and M1R (the orthologs of VACV A27 and L1) and EEV surface proteins B6R and A35R (the orthologs of VACV B5 and A33) have been shown to be the targets of MPXV neutralizing antibodies. In the current study, we recruited 294 volunteers and detected the vaccinia-specific level of residual humoral immunity, including the vaccinia-specific IgG level and neutralizing antibody titer and the cross-antibodies of MPXV A29L, B6R, A35R, and M1R, to evaluate whether remote smallpox vaccination can afford protection against MPXV infection. Our results will provide valuable clues for policy makers to formulate effective vaccination plans against mpox.

## Results

### Humoral immunity from smallpox vaccination

**Fig. 1:**
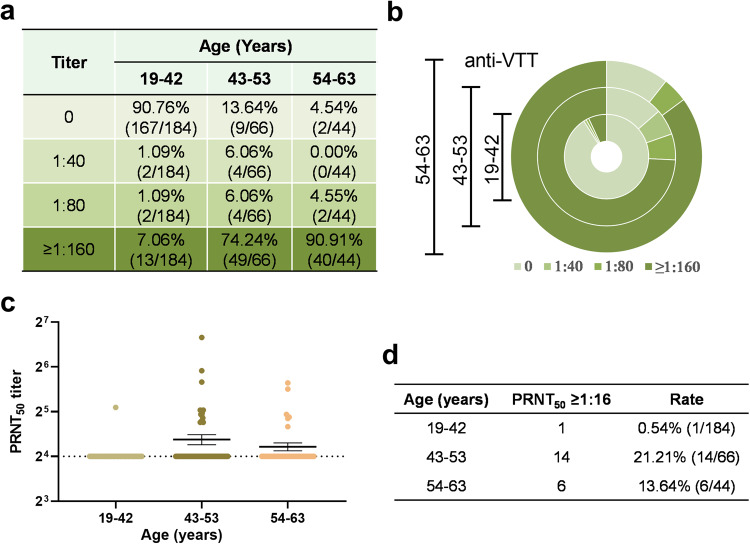
Humoral immunity from smallpox vaccination. Plasma samples from 294 donors (147 males and 147 females) ranging in age from 19–63 years as of 2023 were obtained from Anhui Province in China and divided into three groups according to age: those (*n* = 184, aged 19 to 42 years, as of 2023) born after 1980; those (*n* = 66, aged 43 to 53 years, as of 2023) born between 1970 and 1980, 40–50 years after smallpox vaccination; and those (*n* = 44, aged 54 to 63 years, as of 2023) born between 1960 and 1970, 50-60 years after smallpox vaccination. The level of IgG antibody to the vaccinia virus Tiantan strain (VTT) and the titer of viral neutralizing antibody (VNA) against the VTT were detected by ELISA and plaque reduction neutralization test (PRNT), respectively. The level of VTT-specific serum IgG according to age (**a**, **b**). The neutralizing antibody titers are shown as PRNT_50_ (limit of detection (LOD) = 1:16) according to age (**c**, **d**)

Here, plasma samples from 294 healthy volunteers (147 males and 147 females) ranging in age from 19–63 years as of 2023 were obtained from Anhui Province in China and divided into three groups (aged 19 to 42 years, aged 43 to 53 years, and aged 54 to 63 years) according to age (Table 1). The vaccinia virus Tiantan strain (VTT) was used for the vaccination of Chinese people during the worldwide smallpox prevention campaign.^17^ Therefore, the optical density (OD450) values of VTT-specific IgG were detected by ELISA with plasma dilutions of 1:40, 1:80, and 1:160. The proportion of plasma samples positive for VTT-specific IgG increased with age (9.24% in those aged 19–42 years; 86.36% in those aged 43–53 years; 95.46% in those aged 54–63 years) (Fig. 1a, b). Furthermore, VTT-specific neutralizing antibody (NAb) level was assayed by plaque reduction neutralization test (PRNT). The percentage of people with positive anti-VTT NAb (PRNT50 ≥ 1:16) was 18.18% (20/110) in individuals older than 42 years and slightly decreased from 21.21% (aged 43–53 years) to 13.64% (aged 54–63 years) (Fig. 1c, d), and the detailed anti-VTT NAb titers are shown in Supplementary Table 1. Thus, humoral immunity from the smallpox vaccine in the Chinese population still remains, and VTT-specific NAb level wanes with age.

**Table 1:** The characteristics of 294 healthy volunteers in the First Affiliated Hospital of Anhui Medical University, Hefei City, Anhui Province, China

Age (years)	Number	Sex	Value
19–42 (1981–2004)	184	M	93 (50.54%)
		F	91 (49.46%)
43–53 (1970–1980)	66	M	29 (43.94%)
		F	37 (56.06%)
54–63 (1960–1969)	44	M	25 (56.82%)
		F	19 (43.18%)

*M* male, *F* female

### The plasma antibody level against the mpox virus

**Fig. 2:**
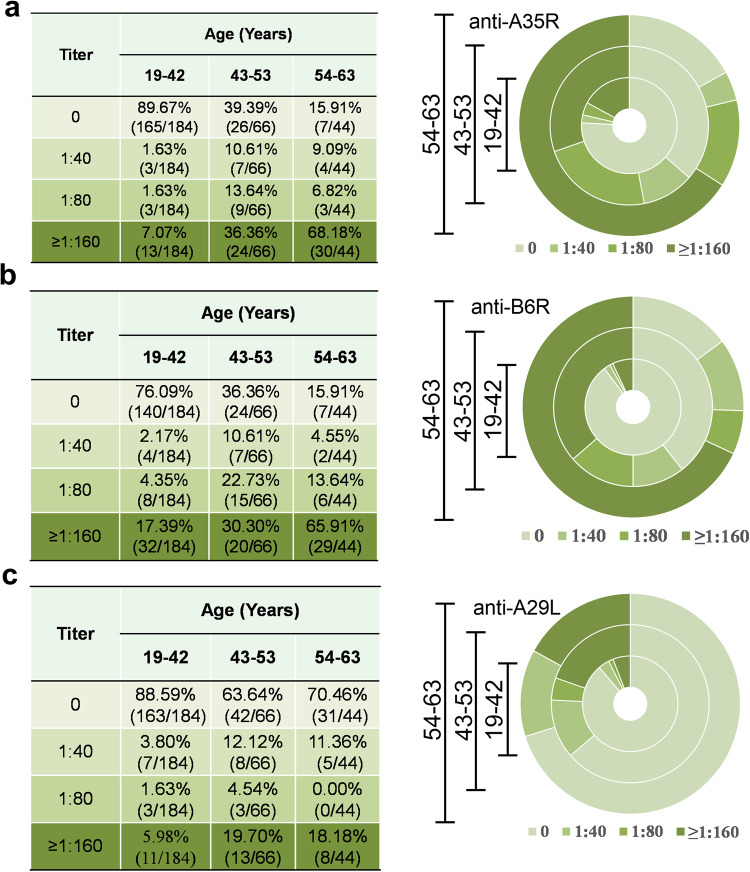
The plasma antibody level against the mpox virus. The level of IgG antibodies against MPXV-specific proteins (A35R, B6R, and A29L) was detected by ELISA. The levels of A35R-, B6R- and A29L-specific serum IgG according to age (**a**, **b**, and **c**)

Many studies have demonstrated that immunity against smallpox has a high protective effect against MPXV.^9,19–21^ Whether immunity from the smallpox vaccine in Chinese people still has cross-protective immunity against MPXV remains unknown. To address this, the IgG levels of 4 MPXV surface proteins, A35R, B6R, A29L and M1R, which are known neutralizing antibody targets (the MPXV orthologs of the VACV *L1R*, *A33R*, *A27L* and *L1* genes, respectively), were detected by ELISA. Firstly, the MPXV-specific antigens A35R, B6R and M1R were expressed in HEK293F cells, and A29L was expressed in *Escherichia coli* BL21. The proteins were confirmed separately by SDS‒PAGE and Western blotting after purification, showing that four purified proteins were successfully obtained (Supplementary Fig. 1). Furthermore, the purified MPXV A35R, B6R, A29L and M1R proteins were used separately to coat the wells of a 96-well flat-bottomed plate to detect the plasma IgG levels at dilutions of 1:40, 1:80, and 1:160. The data shown in Fig. 2 demonstrate that the population with positive anti-A35R/B6R/A29L in pre-1981 (aged 43–53 years; aged 54–63 years) has a much higher proportion than that in post-1981 (aged 19–42 years). In particular, the proportion (targeting A35R or B6R antigen) in those aged 54–63 years was 84.91%. However, the population with positive anti-M1R in pre-1981 (aged 43–53 years; aged 54–63 years) has a higher proportion than that in post-1981 (aged 19–42 years), but the proportion with positive anti-M1R is much lower than that with positive anti-A35R/B6R/A29L (31.82% vs. 60.61%/63.64%/36.36% in aged 43–53 years; 9.09% vs. 84.91%/84.91%/29.54% in aged 54–63 years) (Supplementary Fig. 2). At the same time, the proportion of positive anti-EEV surface proteins (A35R and B6R) is much higher than that of positive anti-IMV surface proteins (A29L and M1R) in the same group (Supplemental Fig. 3). Collectively, the majority of the population pre-1981 who should be immunized with VTT still maintains certain levels of MPXV-specific antibodies, in particular, targeting A35R and B6R antigens.

### Immunity from the smallpox vaccine provides cross-protection against the mpox virus

**Fig. 3:**
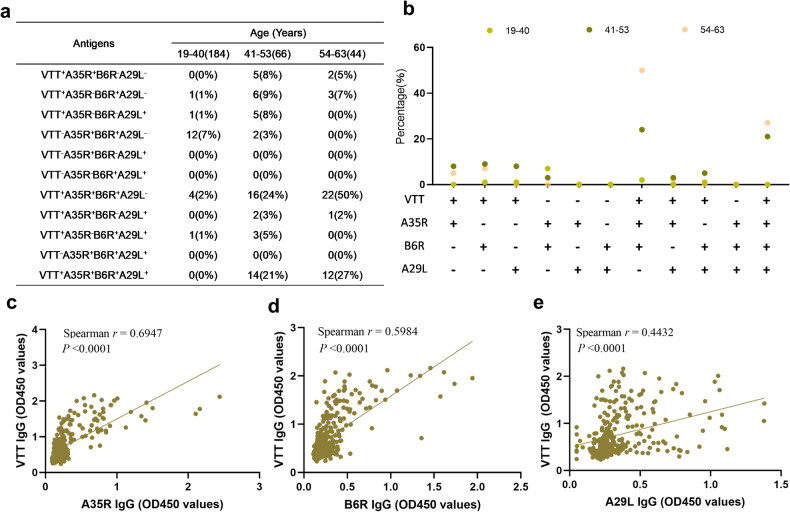
Correlation analysis of VTT-specific IgG and MPXV-specific protein (A35R, B6R, and A29L) IgG. The proportion of plasma samples positive for VTT, A35R, B6R, and A29L antigens by ELISA (**a**, **b**). The correlation between the OD450 values of VTT-specific IgG and A35R-specific IgG, B6R-specific IgG, and A29L-specific IgG with plasma samples diluted 1:40 was separately analyzed (**c**, **d**, and **e**). “+” represents positive; “-” represents negative

Moreover, we calculated the proportion of plasma samples positive for two, three or four antigens of VTT, A35R, B6R, and A29L. As shown in Fig. 3a, b, most of the plasma samples positive for two or three antigens of A35R, B6R, and A29L were positive for VTT. The proportion (VTT^+^A35R^+^B6R^+^A29L^−^) in those aged 54–63 years is as high as 50%. However, if the plasma samples are negative for VTT, the proportion positive for two or three antigens of A35R, B6R, and A29L is 0%, except for VTT^−^A35R^+^B6R^+^A29L^−^. Interestingly, we found that some of the plasma samples were positive for both A35R and B6R antigens and negative for VTT, and the proportion in those aged 19–42 years was higher than that in those aged 43–53 years (7% vs. 3%). This phenomenon reflects that some Chinese people not vaccinated with VTT may have received other orthopoxvirus infections (such as CPXV) and acquired cross-protective immunity against MPXV. Furthermore, we separately analyzed the correlations between the OD450 values of VTT-specific IgG and A35R-specific IgG, B6R-specific IgG, and A29L-specific IgG with plasma samples diluted 1:40, showing a linear correlation (*p* < 0.0001) (Fig. 3c–e). Overall, immunity from the smallpox vaccine may provide some cross-protection against MPXV.

## Discussion

The global mpox outbreak in the shadow of the COVID-19 pandemic has recently drawn more attention. The mpox cases have surged in the last month in China since the whole population has returned to normal life due to the successful control of COVID-19 in Jan. 2023. Although safe and effective vaccines are critically needed to provide protection against MPXV infections, no clinically validated MPXV-specific vaccines are yet available. Smallpox vaccines have been proven to be effective in preventing MPXV infections due to the high genomic homology between the two viruses. However, the eradication of smallpox around the world has made more people vulnerable to MPXV infection, and mpox has gradually become the most significant zoonotic threat to human beings. The immunity level of vaccinated individuals has declined over time, and those younger than 43 years old as of 2023 have not received smallpox vaccination, which may be one of the main reasons for the re-emergence of mpox. To assess the level of residual immunity to smallpox in those people, it is crucial to evaluate the immunological susceptibility to MPXV, providing important clues for revaccination smallpox vaccines and the development of MPXV-specific vaccines in the current mpox outbreak.

Previous studies have demonstrated that virus-specific antibodies are necessary and sufficient to protect against lethal MPXV challenge.^22^ Neutralizing antibody was identified as an important correlate of protection against smallpox. Two studies indicated that high levels of neutralizing antibodies may be associated with protective immunity against smallpox. One of the two demonstrated that subjects who were in contact with smallpox victims possessing vaccinia virus neutralizing titers<1:32 were more susceptible to smallpox infection (3 of 15, or 20% of contacts infected) compared with subjects with antibody titers of ≥1:32 (0 of 127 or <1% of contacts infected).^28^ In another study, the titer of neutralizing Ab against vaccinia virus in human serum was thought to be at least 1:20 to provide protection against smallpox.^29^ However, the available cutoff values for preexisting antibody levels in humans were reported during the period of endemic smallpox circulation, which may not be relevant to the current population. In this study, we evaluated the vaccinia-specific IgG level and neutralizing antibody titers in 294 volunteers, and we found that humoral immunity from the smallpox vaccine in the Chinese population still remains, and VTT-specific NAb level wanes with age. Our results are consistent with previous studies showing that smallpox vaccine-induced immunity wanes with time,^12–14^ and those with low or no VTT-specific antibodies are more susceptible when exposed to MPXV and may need priority vaccination, such people born post-1981 in China.

VTT has played a critical role in the eradication of smallpox in China.^25^ Therefore, the titers of VTT-specific IgG were detected, and we found that the proportion of plasma samples positive for VTT-specific IgG increased with age, showing that the majority of the Chinese population in pre-1981 still retained the binding antibody response to vaccinia after vaccination for 42 or more years. Furthermore, the VTT-specific NAbs were also positive (PRNT50 ≥ 1:16) in 18.18% (20/110) of individuals older than 42 years, but the positive rate of anti-VTT NAb was slightly lower in people aged 54–63 years than in those aged 43–53 years. Thus, the current populations in China who received the smallpox vaccine decades ago still retain some humoral immunity against VTT, and VTT-specific NAb level declines with age.

Due to genetic and antigenic homology, smallpox vaccines have historically been used to prevent or reduce the severity of mpox.^25,30^ However, with the decline in VTT-specific antibody response, whether residual immunity among Chinese people could still provide cross-protection against mpox remains unknown. A number of MPXV envelope proteins, such as MIR, A35R, B6R, and A29L (the MPXV orthologs of the VACV *L1R*, *A33R*, and *A27L* genes, respectively), have been identified as targets of neutralizing antibodies.^31^ In this study, we detected the IgG levels of MPXV surface proteins MIR, A35R, B6R, and A29L and found that the population with positive anti-A35R/B6R/A29L in pre-1981 who should have received smallpox vaccination has a much higher positive proportion than that in people younger than 43 years old who probably have not been vaccinated. In particular, people 54–63 years old have a high proportion of antibodies targeting the A35R or B6R antigen. Therefore, the majority of the smallpox vaccine-immunized population still maintains certain levels of MPXV-specific antibodies, especially those targeting the A35R and B6R antigens. Moreover, the plasma samples positive for VTT were also positive for two or three antigens of A35R, B6R, and A29L, and the titers of VTT-specific IgG and A35R-specific IgG, B6R-specific IgG, and A29L-specific IgG were linearly correlated (*p* < 0.0001). However, the positive rate of anti-M1R was relatively low in all subjects, and the proportion with positive anti-A29L and anti-M1R is much higher than that with positive anti-A35R and anti-B6R in the 54- to 63-year-old group, which is contrary to the level of VTT-specific NAbs. We speculated that the binding abilities of A29L and MIR were slightly higher than those of A35R and B6R antigens,^32^ and as the anti-A29L and MIR levels declined, the NAb levels also waned. Interestingly, some younger people aged 19–42 years were VTT negative but were positive for both A35R and B6R antigens, and the proportion in those was higher than that in people aged 43–53 years. This may be due to other orthopoxvirus infections (such as CPXV) and acquired cross-protective immunity against MPXV. The above phenomenon also suggests that people immunized with the smallpox vaccine are less likely to be infected with other poxviruses.

Although this remaining immunity from smallpox vaccination may not provide full protection against MPXV infection, it is likely to decrease the probability of severe and fatal disease and relieve clinical symptoms after infection. However, to acquire more complete information regarding the immunity level against MPXV among the Chinese population, further investigations are required in terms of increasing the sample size and evaluating the live MPXV neutralizing antibody levels and the cellular immune response after vaccination against smallpox. Furthermore, a global mpox outbreak is currently primarily affecting gay, bisexual, and other men who have sex with men,^33^ which are high-risk populations. The immunity level of high-risk populations to MPXV is required to be investigated.

In summary, our results indicate that most Chinese populations still maintain VTT-specific IgG antibody for 42 or more years after smallpox vaccination and could provide mpox virus-specific IgG antibodies. Our findings contribute to prioritizing the anti-MPXV strategy and prevention in the population under 43 years of age, especially high-risk populations, such as gay, bisexual, or other men who have sex with men, as well as immunocompromised subjects.

## Materials and methods

### Participants

The study was reviewed and approved by the Ethics Committee of The First Affiliated Hospital of Anhui Medical University (approval No. PJ2023-03-39). Two hundred ninety-four volunteers participated in the study from Anhui Province in China ranging in age from 19–63 years as of 2023 and were divided into three groups according to age. The first group (n = 184) was generally younger (aged 19 to 42 years) and was born after smallpox vaccination in post-1981. The second group (*n* = 66) was aged 43–53 years with a history of smallpox vaccinations between 1970 and 1980. The third group (*n* = 44) was aged 54–63 years. The detailed characteristics of 294 healthy volunteers are shown in Table 1.

### Viruses and cells

Vaccinia virus (VACV) Tiantan strain (VTT) was grown in Vero cells by using a multiplicity of infection of 0.01 and harvested at 48 h post-infection. Cells were lysed by 3 freeze/thaw cycles. The plaque-forming unit (PFU) of viral stocks was titrated on Vero cells by a plaque-forming assay using crystal violet staining. The virus was inactivated with 0.025% β-propiolactone (v/v) before purification by ultracentrifugation through a 36% sucrose cushion.^34^ African green monkey kidney (Vero) cells were grown in high-glucose Dulbecco’s modified Eagle’s medium (DMEM) supplemented with 10% fetal bovine serum (FBS; Shanghai VivaCell Biosciences Ltd, Shanghai, China) and 1% penicillin/streptomycin (Thermo Fisher Scientific) at 37 °C with 5% CO_2_. Human embryonic kidney 293F (HEK293F) cells were cultured in HEK293 Cell Complete Medium (Sino Biological Inc., Beijing, China) supplemented with 1% penicillin/streptomycin.

### Recombinant MPXV A35R, B6R, A29L, and M1R proteins expression and purification

MPXV-specific antigens A35R, B6R, A29L, and M1R (GenBank: NC_063383.1) were expressed and purified to detect the IgG antibody titers in plasma. Briefly, the codon-optimized sequences of the MPXV *A35R, B6R*, and *M1R* genes were synthesized and cloned into the pCDNA3.1 vector (Life Technologies) with a His-tag. The recombinant plasmids were transfected into HEK293F cells, and the A35R, M1R, and B6R proteins were expressed and purified. The proteins were confirmed by Coomassie-stained sodium dodecyl sulfate–polyacrylamide gel electrophoresis (SDS‒PAGE) and Western blotting. The *A29L* gene was also synthesized and cloned into the prokaryotic expression vector PET-28a (+) with a His-tag, and the expression plasmid was transformed into *Escherichia coli* BL21(DE3) expression host cells (Novagen) to express the recombinant A29L protein. The A29L protein is a soluble protein and was purified from cell lysates. The purified quality of the proteins was confirmed by SDS‒PAGE, and the expressed A35R, B6R, A29L, and M1R proteins were identified by Western blotting with anti-A35R antibody (R392a3, OkayBio, China), anti-B6R antibody (R401c8, OkayBio, China), anti-A29L antibody (R396t5, OkayBio, China), and anti-M1R antibody (R403k5, OkayBio, China), respectively.

### Enzyme-linked immunosorbent assay (ELISA)

ELISA was used to detect anti-vaccinia antibody and MPXV cross-antibody. Plasma samples were heat-inactivated for 30 min at 56 °C. The presence of vaccinia-specific IgG antibody and MPXV cross-antibody were tested as previously described.^34^ Briefly, VTT (equivalent concentrations: 1 × 10^7^ PFU/mL) or MPXV A35R, B6R, A29L, and M1R proteins were used to coat the wells of a 96-well flat-bottomed plate. Plasma dilutions of 1:40, 1:80, and 1:160 were placed in antigen-coated wells and incubated at 37 °C for 1 h. The plates were washed, horseradish peroxidase-conjugated anti-human IgG was added, and the plates were again incubated for 1 h at 37 °C. After incubation, 100 µL TMB (3,3',5,5'-tetramethylbenzidine) per well was added. The color development was stopped by adding H_2_SO_4_. Optical density values were read at 450 nm using an ELISA plate reader (Thermo Fisher Scientific, Waltham, MA, USA).

### Neutralization antibody titer assay

VTT-specific neutralizing antibody titers were measured by the plaque reduction neutralization titer (PRNT) assay as previously described.^35,36^ Heat-inactivated plasma samples were serially diluted (starting at 1:16) and incubated with 100 PFU of the VTT for 1 h at 37 °C, and then the virus-plasma mixtures were added to Vero cell monolayers seeded in 24-well plates and incubated for 1 h at 37 °C. After adsorption, culture medium containing 0.9% carboxymethyl cellulose was added to each well to allow plaque formation. After culturing at 37 °C for 40 h, cell monolayers were stained with 1% crystal violet dissolved in 70% ethanol. Monolayers were rinsed with water, and the plaques were counted.

### Statistical analysis

All statistical analyses were performed using GraphPad Prism 9.0 (GraphPad Software, La Jolla, CA, USA). The PRNT_50_ titers were converted to natural logarithms to normalize their distributions. Data are represented as the mean ± SEM. Linear correlation was evaluated by Spearman’s correlation coefficient. *p*-values < 0.05 (two-tailed) were considered statistically significant.

### Supplementary information

Supplementary Materials

## Data Availability

All data are available upon reasonable request to the corresponding author.

## References

[1] Petersen E (2019). Human Monkeypox: epidemiologic and clinical characteristics, diagnosis, and prevention. Infect. Dis. Clin. North Am..

[2] Breman JG (1980). Human monkeypox, 1970-79. Bull. World Health Organ..

[3] Bunge EM (2022). The changing epidemiology of human monkeypox-A potential threat? A systematic review. PLoS Negl. Trop. Dis..

[4] Costello V (2022). Imported Monkeypox from International Traveler, Maryland, USA, 2021. Emerg. Infect. Dis..

[5] Hobson, G., et al. Family cluster of three cases of monkeypox imported from Nigeria to the United Kingdom, May 2021. *Euro. Surveill.* **26**, 2100745 (2021). 10.2807/1560-7917.ES.2021.26.32.2100745 PMC8365177 34387184

[6] Erez N (2019). Diagnosis of imported Monkeypox, Israel, 2018. Emerg. Infect. Dis..

[7] WHO*. WHO Director-General’s statement at the press conference following IHR Emergency Committee regarding the multi-country outbreak of monkeypox—23 July 2022*. https://www.who.int/director-general/speeches/detail/who-director-general-s-statement-on-the-press-conference-following-IHR-emergency-committee-regarding-the-multi--country-outbreak-of-monkeypox--23-july-2022. (WHO, 2022).

[8] CDC. *2022 Global Map & Case Count*. https://www.cdc.gov/poxvirus/mpox/response/2022/world-map.html. (CDC, 2023).

[9] Fine PE, Jezek Z, Grab B, Dixon H (1988). The transmission potential of monkeypox virus in human populations. Int. J. Epidemiol..

[10] Henderson DA (2011). The eradication of smallpox–an overview of the past, present, and future. Vaccine.

[11] Kim SH (2007). The persistence of humoral and cellular immunities more than three decades after smallpox vaccination. Clin. Microbiol. Infect.

[12] Amanna IJ, Slifka MK, Crotty S (2006). Immunity and immunological memory following smallpox vaccination. Immunol. Rev..

[13] Mooi FR, Van Der Maas NA, De Melker HE (2014). Pertussis resurgence: waning immunity and pathogen adaptation—two sides of the same coin. Epidemiol. Infect..

[14] Heffernan JM, Keeling MJ (2009). Implications of vaccination and waning immunity. Proc. Biol. Sci..

[15] Shchelkunov SN (2013). An increasing danger of zoonotic orthopoxvirus infections. PLoS Pathog.

[16] Nguyen PY (2021). Reemergence of Human Monkeypox and declining population immunity in the context of urbanization, Nigeria, 2017-2020. Emerg. Infect. Dis..

[17] Nalca A, Zumbrun EE (2010). ACAM2000: the new smallpox vaccine for United States Strategic National Stockpile. Drug Des. Devel. Ther..

[18] Rizk JG (2022). Prevention and treatment of Monkeypox. Drugs.

[19] Xiang Y, White A (2022). Monkeypox virus emerges from the shadow of its more infamous cousin: family biology matters. Emerg. Microbes Infect..

[20] FDA. *FDA Approves First Live, Nonnon-replicating Vaccine to Prevent Smallpox and Monkeypox*. https://www.fda.gov/news-events/press-announcements/fdaapproves-first-live-non-replicating-vaccine-prevent-smallpox-and-monkeypox. (FDA, 2019).

[21] Yang X (2022). The life cycle of Hyalomma scupense (Acari: Ixodidae) under laboratory conditions. Ticks Tick Borne Dis..

[22] Thy M (2022). Breakthrough infections after postexposure vaccination against Mpox. N. Engl J. Med..

[23] Turner M (2022). Monkeypox in patient immunized with ACAM2000 smallpox vaccine During 2022 Outbreak. Emerg. Infect. Dis..

[24] Poland GA, Kennedy RB, Tosh PK (2022). Prevention of monkeypox with vaccines: a rapid review. Lancet Infect. Dis..

[25] Gong Q, Wang C, Chuai X, Chiu S (2022). Monkeypox virus: a re-emergent threat to humans. Virol. Sin..

[26] Yang L (2023). Immunization of mice with vaccinia virus Tiantan strain yields antibodies cross-reactive with protective antigens of monkeypox virus. Virol. Sin..

[27] Panchanathan V, Chaudhri G, Karupiah G (2008). Correlates of protective immunity in poxvirus infection: where does antibody stand?. Immunol. Cell Biol..

[28] Mack TM, Noble J, Thomas DB (1972). A prospective study of serum antibody and protection against smallpox. Am. J. Trop. Med. Hyg..

[29] Sarkar JK, Mitra AC, Chakravarty MS (1972). Relationship of clinical severity, antibody level, and previous vaccination state in smallpox. Trans. R. Soc. Trop. Med. Hyg..

[30] Jezek Z (1986). Human monkeypox: a study of 2,510 contacts of 214 patients. J. Infect. Dis..

[31] Davies DH (2005). Profiling the humoral immune response to infection by using proteome microarrays: high-throughput vaccine and diagnostic antigen discovery. Proc. Natl Acad. Sci. USA.

[32] Gao F (2023). Cross-reactive immune responses to monkeypox virus induced by MVA vaccination in mice. Virol. J..

[33] Delaney KP (2022). Strategies adopted by gay, bisexual, and other men who have sex with men to prevent monkeypox virus transmission—United States, August 2022. MMWR Morb. Mortal Wkly Rept..

[34] Hammarlund E (2003). Duration of antiviral immunity after smallpox vaccination. Nat. Med..

[35] Hooper JW, Custer DM, Schmaljohn CS, Schmaljohn AL (2000). DNA vaccination with vaccinia virus L1R and A33R genes protects mice against a lethal poxvirus challenge. Virology..

[36] Mucker EM (2022). A nucleic acid-based orthopoxvirus vaccine targeting the vaccinia virus L1, A27, B5, and A33 proteins protects rabbits against lethal rabbitpox virus aerosol challenge. J. Virol..

