# Supplementary material for: Duration of humoral immunity from smallpox vaccination and its cross-reaction with Mpox virus
Source: Signal Transduct Target Ther. 2023 Sep 15;8:350. doi: 10.1038/s41392-023-01574-6 (PMC10502045; doi:10.1038/s41392-023-01574-6)
Supplement: Supplementary file 1 — Supplementary Materials [file 41392_2023_1574_MOESM1_ESM.docx]

Supplementary Materials for

Duration of Humoral Immunity from Smallpox Vaccination and its Cross-reaction with Mpox Virus

Entao Li^1,2*^, Xiaoping Guo^2*^, Dongxiang Hong^2^, Qizan Gong^2^, Wenyu Xie^2^, Tingting Li^3^, Jian Wang^2✉^, Xia Chuai^4✉^, Sandra Chiu^1,2,5,6✉^

**Correspondence to:**

Sandra Chiu, qiux@ustc.edu.cn; Xia Chuai, chuaixiahb@126.com; Jian Wang, ustcwj@ustc.edu.cn;

**This PDF file includes:**

Supplemental Table 1

Supplemental Figures 1, 2, and 3

**Supplemental Table 1**

**Table S1** The neutralizing antibody titers against the vaccinia virus Tiantan strain

| Age group (years) | Sample No. | Birth year | Titer |
| --- | --- | --- | --- |
| 19-42 | 197 | 1994 | 1:34 |
| 43-53 | 250 | 1980 | 1:100 |
|  | 278 | 1978 | 1:31 |
|  | 292 | 1977 | 1:30 |
|  | 141 | 1976 | 1:31 |
|  | 289 | 1976 | 1:51 |
|  | 270 | 1975 | 1:33 |
|  | 293 | 1975 | 1:29 |
|  | 300 | 1975 | 1:27 |
|  | 259 | 1973 | 1:32 |
|  | 277 | 1973 | 1:33 |
|  | 298 | 1973 | 1:28 |
|  | 149 | 1971 | 1:60 |
|  | 258 | 1971 | 1:27 |
|  | 287 | 1971 | 1:28 |
| 54-63 | 291 | 1968 | 1:45 |
|  | 126 | 1967 | 1:29 |
|  | 268 | 1965 | 1:31 |
|  | 254 | 1963 | 1:50 |
|  | 296 | 1963 | 1:25 |
|  | 265 | 1961 | 1:29 |

**Supplemental Figure 1**

**
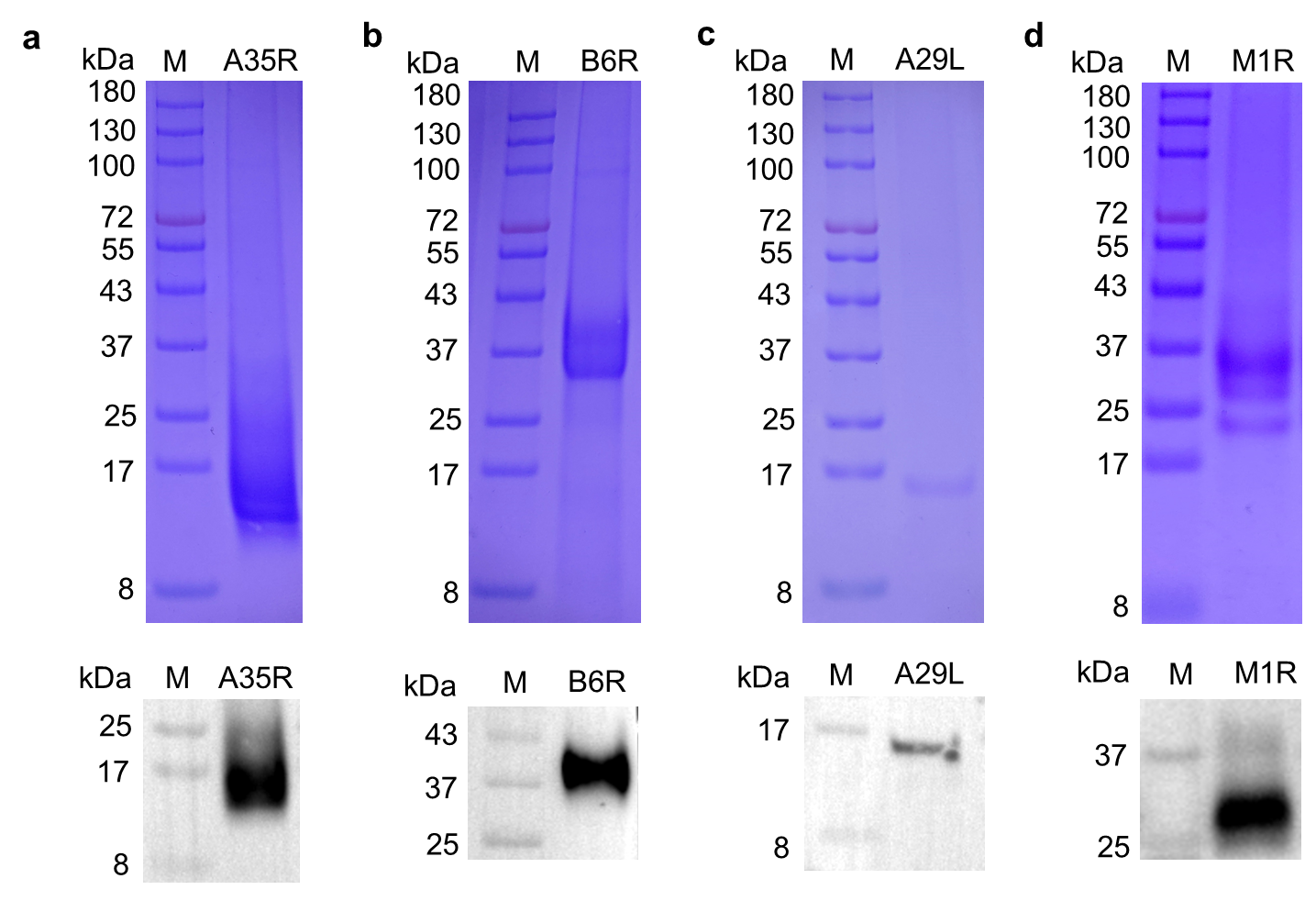
**

**Figure S1. The MPXV A35R, B6R, A29L, and M1R proteins were identified by SDS‒PAGE and Western blotting.** M: marker.

**Supplemental Figure 2**


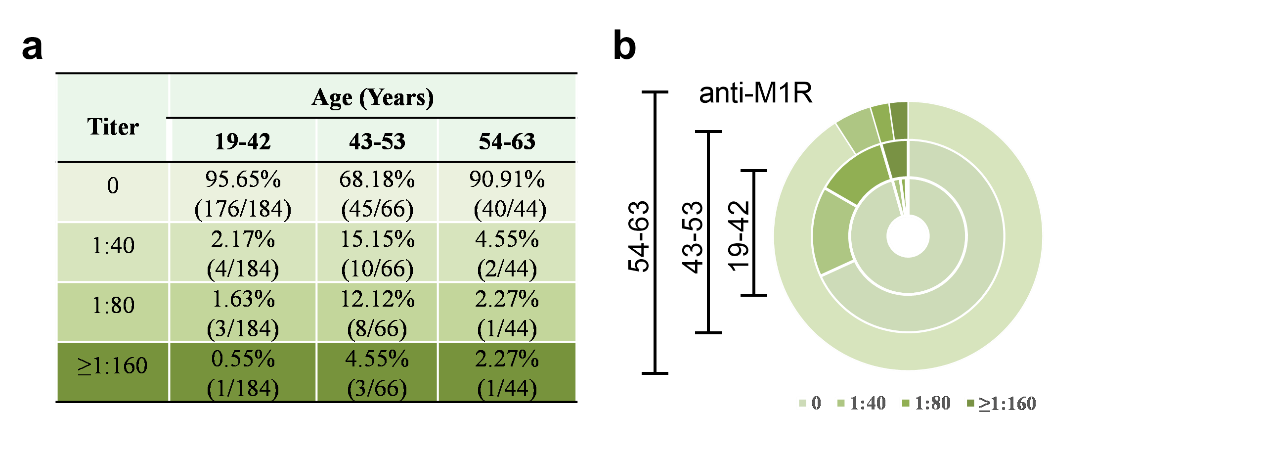


**Figure S2.** The plasma antibody level against the MPXV-specific M1R protein. The level of IgG antibody against M1R was detected by ELISA. The level of M1R-specific serum IgG according to age (**a and b**).

**Supplemental Figure 3**


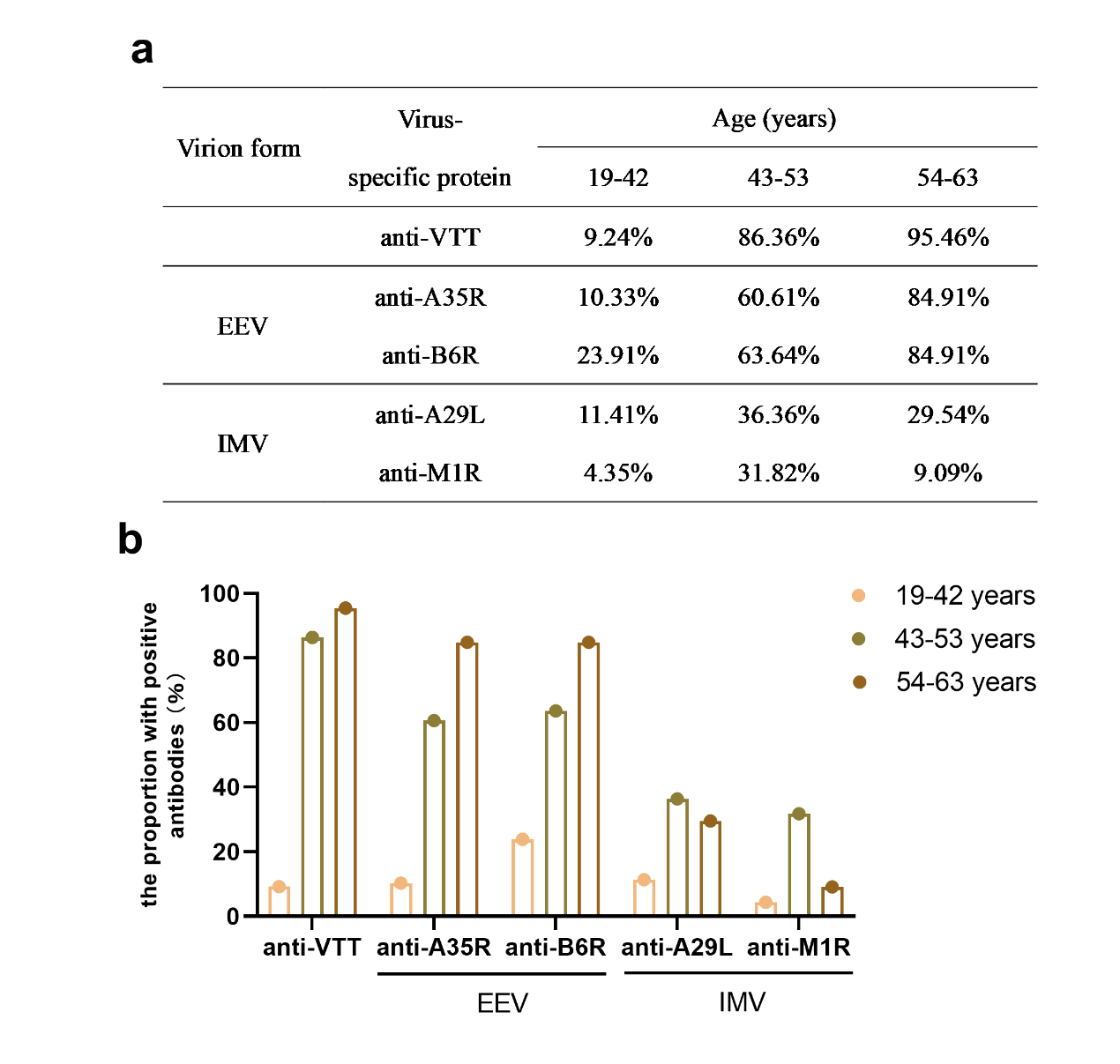


**Figure S3.** The proportion of positive anti-VTT or anti-4 MPXV surface protein antibodies in different populations. To assess the level of immunity to the vaccinia virus Tiantan strain (VTT) in individuals and evaluate their immunological susceptibility to MPXV, the level of IgG antibody to the VTT and MPXV-specific proteins (A35R, B6R, A29L, and M1R) was detected by ELISA. The proportion with positive anti-VTT or 4 MPXV surface proteins in individuals with plasma IgG levels at dilutions of 1:40 (**a and b**).
